# Teleophthalmology for triage and management of corneal pathologies at vision centres: a prospective observational service evaluation study in north India

**DOI:** 10.3389/fopht.2026.1867442

**Published:** 2026-06-23

**Authors:** Nikunj Vinodbhai Patel, Meenal Kataria, Manvi Aggarwal, Nagma Ansari, Atanu Majumdar, Shalinder Sabherwal, Birendra Singh, Umang Mathur, Manisha Acharya

**Affiliations:** 1 Department of Cornea, Dr. Shroff’s Charity Eye Hospital, Delhi, India; 2 Department of Public Health, Dr. Shroff’s Charity Eye Hospital, Delhi, India

**Keywords:** cornea, referral, resource-limited, rural India, teleophthalmology, vision centre

## Abstract

**Purpose:**

To assess the feasibility of teleophthalmology for triage and management of corneal diseases at vision centres (VCs) in rural and semi-urban North India.

**Methods:**

All patients presenting to the VCs with corneal pathology who underwent teleconsultation for corneal disease during the study period (March-September 2024) were included in the study. Those who reported before or after the study period were not included. After registration and preliminary examination at the VC, unstained and stained images of patients with corneal pathologies were sent to an in-training cornea fellow at the tertiary centre via a web-based application. Management at the VC was carried out by vision technicians under specialist supervision; acute cases received preliminary emergency care and were referred to a secondary care hospital. Non-acute pathologies were managed at the primary level or referred as needed. Primary outcomes were the proportion of patients managed at VCs versus those referred, and compliance with referrals. Secondary outcomes were factors influencing referral compliance and reporting timelines for emergency and non-emergency cases.

**Results:**

Of 4, 825 patients with corneal disease, 2, 022 (41.91%) were managed at VCs, while 2, 803 (58.09%) were referred. Hot (acute/emergency) corneal cases comprised 1, 852; cold (non-acute/non-emergency) cases comprised 2, 973. Overall referral compliance was 27.58% (772/2, 803 referred cases). Regression analysis identified significant predictors of referral uptake, including proximity to hospitals and longer-established centres (p<0.001 for most associations). Acute cases and those from centres more than one year old were more likely to reach the hospital after referral.

**Conclusions:**

Teleconsultation provided by specialist ophthalmologists enabled local triage and management of corneal diseases at the primary level, reduced unnecessary referrals, and helped counsel patients in emergency conditions. This model can be adopted by other ocular specialities and strengthen primary care in resource-constrained areas.

## Introduction

As per the National Programme for Control of Blindness and Visual Impairment (NPCBVI) report (2015-2019), corneal opacity is the second most common cause of blindness (8.2%) after cataract in the population more than 50 years of age in India. However, it becomes the most common cause of blindness in the population under 50 years of age, accounting for 37.5% of cases. Overall, the percentage of blindness in the population more than 50 was higher in rural parts as compared to urban areas (2.14% v/s 1.80%) (1).

There is a significant disparity in healthcare infrastructure and manpower between urban and rural India, despite 63% of the population living in rural areas (2, 3). The gap is even wider for specialized services like Ophthalmology due to a shortage of doctors in rural regions (4). Because of limited access, patients tend to delay treatment or miss follow-up appointments. According to the National Sample Survey Office’s (NSSO) 65^th^ round on tourism, it was found that 86% of all trips for medical purposes are made by rural Indians, with poorer individuals spending proportionally more (5). A large population in North India resides in rural areas and is engaged in agricultural activities (4, 6).

Enhancements in rural eye care require a comprehensive approach that emphasizes accessibility, affordability, and consistent quality (7). Providing primary care at vision centres (VCs) can not only reduce the disease burden but also lessen the need for highly skilled personnel in remote areas (8). Teleophthalmology helps bridge this gap and has been shown to decrease both healthcare infrastructure costs and financial burdens for patients. It also lowers the carbon footprint and offers notable environmental advantages (9). As a result, VCs are increasingly becoming the preferred model for delivering primary eye care, with many organizations integrating telemedicine into their services (10).

Komal et al. studied the effectiveness of teleophthalmology in managing corneal disorders at VC, where they considered only a single VC. They found this model to be highly effective in preventing ocular morbidity caused by corneal conditions, especially in vulnerable populations of rural villages, and recommended replicating the VC-based teleophthalmology system to provide universal eye health in a cost-effective manner (11).

With a well-developed primary eye care network consisting of VCs near secondary centres offering cornea services, and tertiary care available through teleophthalmology, we aimed to study the role of teleophthalmology in managing corneal pathologies at VCs. In our network, cornea teleconsultations are provided by in-training cornea fellows (CF), making it relevant to evaluate the effectiveness of this model within our setting.

To our knowledge, this is the first study investigating the use of a teleophthalmology program focused on the management of corneal pathologies in North India.

## Methods

This was a prospective descriptive study conducted at the cornea and anterior segment department of a tertiary care centre in North India from March 1, 2024, to September 30, 2024.

Dr. Shroff’s Charity Eye Hospital (SCEH) network includes one tertiary care centre located in New Delhi, eight secondary care centres across three states in North India (Uttar Pradesh, Rajasthan, and Uttarakhand), and 112 VCs. Each VC serves a rural population of 50, 000 people and is connected to a secondary care centre for patient referrals. Each VC is staffed by a vision technician (VT) and a community health worker (CHW). VTs are posted at their respective VCs after undergoing two years training at a higher centre and are supervised for another one year before being given independent posting. They are trained in general eye examination including refraction, intraocular pressure measurement, corneal staining, superficial foreign body removal, basic smartphone imaging, teleconsulting via software. Annual training is conducted to review standard operating procedures for teleconsultation.

All patients presenting to the VC with corneal pathologies were included in the study. Patients who did not undergo tele-consultation and those who reported to the VCs before and after the study period were excluded. Written informed consent was obtained from patients at the time of registration; no separate consent was required for teleconsultation.

### Operational framework of telemedicine: registration, initial evaluation at VCs, and specialist consultation

All VCs within the hospital network use an in-house developed web-based software platform called the Vision Centre Management System (VCMS) to manage patient data and facilitate teleconsultations.

**Figure 1:**
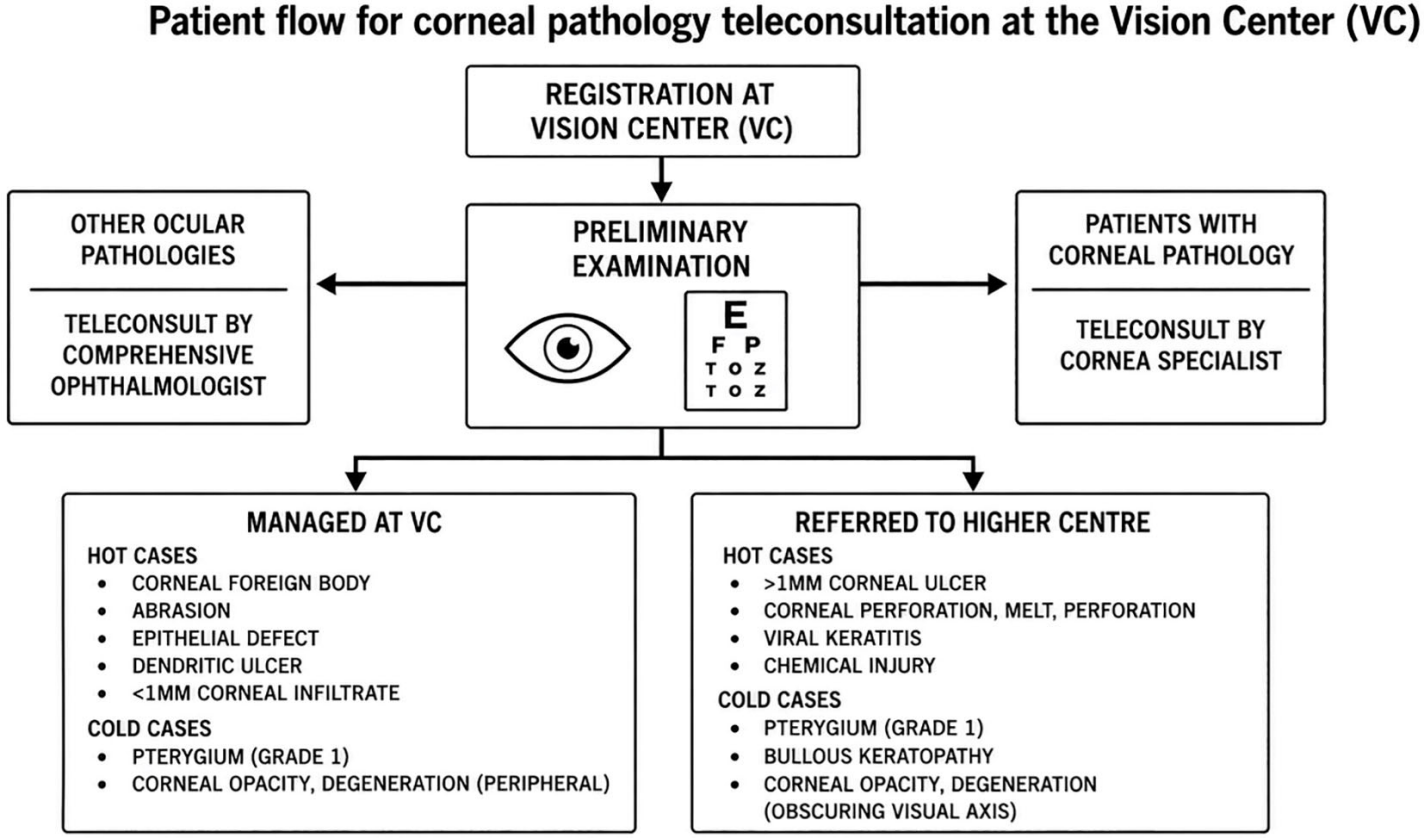
Schematic representation of the teleconsultation framework.

When a patient visits a VC, they are first registered in the VCMS software by the VT (**Figure 1**). The patient’s demographic details, medical and ocular history—including chief complaints, present and past illnesses, systemic and family history, and any prior surgeries—are entered into the system.

Following this, a thorough preliminary examination is performed. This includes slit lamp examination, visual acuity assessment, objective and subjective refraction, and the final spectacle prescription.

**Figure 2:**
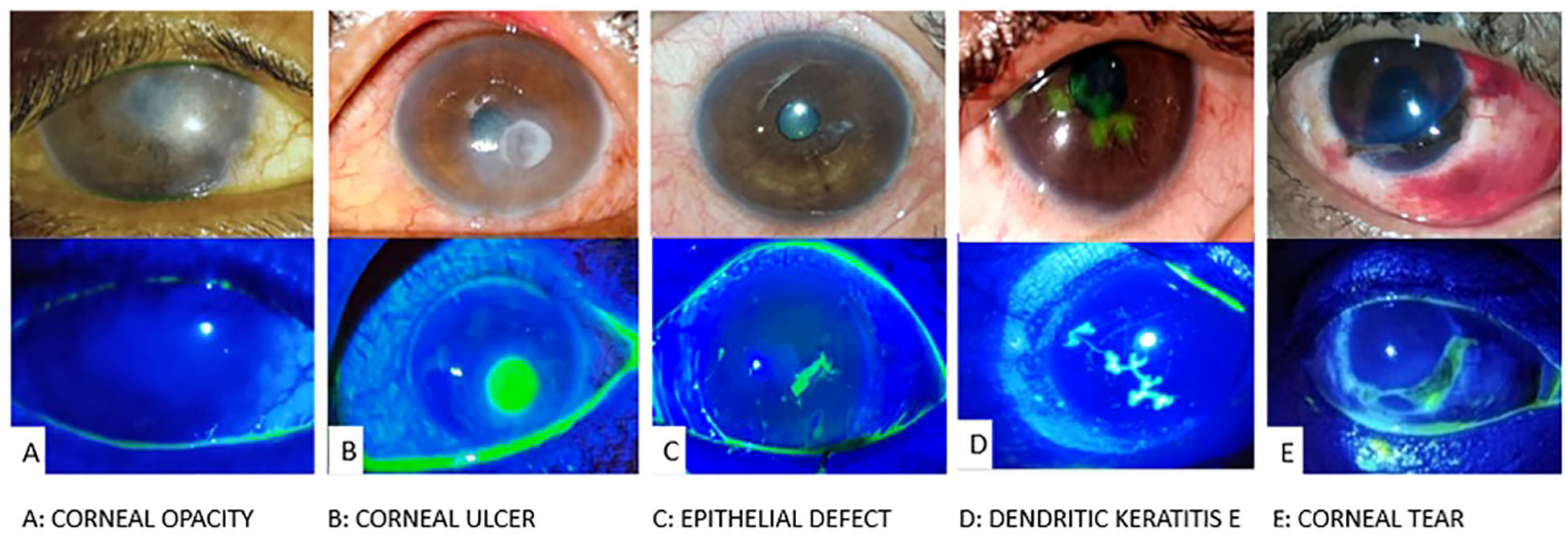
Unstained and fluorescein-stained images clicked by vision technician for the purpose of teleconsultation. **(A)** Corneal opacity, **(B)** corneal ulcer, **(C)** epithelial defect, **(D)** dendritis keratitis, **(E)** corneal tear.

Images of the anterior segment of the eye are captured using a smartphone. If any corneal pathology is suspected, additional fluorescein-stained images are taken under cobalt blue light (**Figure 2**). All images are uploaded directly into the patient’s file on the VCMS software.

Based on the clinical findings, the case is triaged. If a corneal pathology is suspected, the case is referred to a cornea specialist via teleconsultation. All other cases are reviewed by a comprehensive ophthalmologist.

Cornea fellows (CF) at the base hospital, who had been in the program for at least six months, were credentialed to perform teleconsultations. CF reviews the clinical details and anterior segment images in the VCMS software to establish a diagnosis. If additional patient history or more images are needed to help with diagnosis or treatment planning, the CF works with the VT at the respective VC. When necessary, the vision technician arranges a real-time video consultation between the patient and the CF at the command centre. During the video call, the patient and the team discussed the disease and possible treatments in detail.

The cases were then classified as ‘hot’ cases (acute/emergency) and ‘cold’ cases (non-acute/non-emergency). A management plan was formulated accordingly. Hot cases include those requiring immediate intervention, such as corneal foreign body, epithelial defect, corneal ulcer, and corneal tear. Cold cases include non-emergency conditions like pterygium, corneal opacity, and corneal degeneration.

Once the patient’s file is reviewed by the CF, a decision is made regarding the appropriate course of management—whether the patient can be treated at the VC or requires referral to a higher centre. For patients who can be managed at the VC, the CF enters the treatment prescription, follow-up date, and any additional advice related to the patient’s management into the VCMS software.

The vision technician then provides the patient with a printed copy of the prescription and clearly explains the treatment plan, including the prescribed medications and how to take them correctly. The technician also shares the follow-up appointment date and any specific instructions or advice from the specialist. Emphasis is placed on the importance of following the treatment and attending follow-up visits to achieve the best results.

For patients requiring referral, the CF explains the reason and its importance through a video consultation. For urgent cases, preliminary treatment is advised, followed by referral to the appropriate secondary centre. The patient is counselled about the urgency of their condition and the critical importance of timely follow-up and treatment.

In non-emergency cold cases, no initial treatment was administered. The patient was informed about the diagnosis, given a referral letter, and advised to visit the secondary centre for further evaluation and management at the earliest convenience.

Referred patients presenting at the secondary centres were registered in the Electronic Medical Records (EMR) system. Their demographic information, clinical history, and preliminary examination findings were documented. Subsequent evaluation and management were conducted by the cornea consultant at the respective secondary centre.

Referral compliance was defined as a patient registering at any secondary/tertiary centre within the study period. VC establishment age was divided into categories: <1 year, 1–3 years, 3–5 years, 5–10 years, and > 10 years. Travel time was measured as the Google Maps distance to the nearest secondary centre and categorised into <30 minutes, 30–60 minutes and > 60 minutes.

### Data collection

Patient data for individuals who underwent teleconsultation were retrieved from the VCMS, while data for those who reported to the secondary centres were extracted from the EMR system. A unified database was created by linking the records of referred patients with those of patients who subsequently reported to the centres. Data analysis was performed using Microsoft Excel (version 2024).

### Statistical analysis

Patient-level data retrieved from the VCMS and EMR were directly exported to the R platform (version 4.5.0) for statistical analysis. Descriptive statistics summarized demographic characteristics and clinical diagnoses. Categorical variables were presented as counts and percentages. To identify factors associated with reporting to higher-level care, a multivariable binary logistic regression model was fitted. Independent variables included patient age, gender, nature of diagnosis (acute vs. non-acute), travel time to the hospital, VC staff characteristics, and the duration since the centre was established. A p-value of less than 0.05 was considered statistically significant.

The study adhered to the tenets of the Declaration of Helsinki and was approved by the Institutional Ethics Committee.

## Results

From March to September 2024, a total of 20, 308 patients received teleconsultations across the SCEH network. Of these, 4825 patients (23.75%) were diagnosed with corneal pathologies.

Of these 4825 patients, 1852 (38.38%) presented with painful acute corneal disorders. The most common painful acute corneal disorder was corneal ulcer (n = 658), and the most common non-acute corneal disorder was pterygium (n = 1062).

Out of the 4, 825 patients, 2, 022 (41.91%) were managed at the VCs, while 2, 803 (58.09%) were referred to secondary centre hospitals within our hospital network. Among the 2, 022 patients treated at the VCs, 929 (45.94%) presented with acute corneal conditions, and 1, 093 (54.06%) with non-acute corneal conditions.

**Table 1** presents the distribution of corneal pathologies, showing patient counts across diagnostic categories as well as referral and reporting trends to secondary care centres.

**Table 1:** Details of patients managed through teleconsultation.

Diagnosis	Total cases seen (n)	Managed at VC (n)	Referred to SC (n)	Percent among the total cases referred (%)	Number of patients reaching SC after referral (n)	Proportion reaching SC after referral (%)
Cold Cases						
Pterygium	1062	190	872	46.38%	204	23.39%
Corneal Opacity	1046	177	869	46.22%	207	23.82%
Bullous Keratopathy	42	2	40	2.13%	13	32.50%
Dry Eye	658	619	39	2.07%	6	15.38%
Others	165	105	60	3.19%	14	23.33%
Cold Cases (TOTAL)	2973	1093	1880	100%	444	23.62%
Hot Cases						
Ulcer	658	103	555	60.13%	187	33.69%
Epithelial Defect	419	324	95	10.29%	15	15.79%
Viral Keratitis	109	23	86	9.32%	57	66.28%
Foreign Body	448	380	68	7.37%	19	27.94%
Chemical Injury	49	18	31	3.36%	10	32.26%
Dendritic Keratitis	46	22	24	2.60%	11	45.83%
Corneal Perforation	20	0	20	2.17%	11	55.00%
Corneal Abrasion	70	57	13	1.41%	2	15.38%
Corneal Tear	12	0	12	1.30%	6	50.00%
Corneal Melt	11	0	11	1.19%	9	81.82%
Herpes Zoster Ophthalmicus	10	2	8	0.87%	2	25.00%
Hot Cases (TOTAL)	1852	929	923	100%	329	35.64%

• VC, vision centre, SC, secondary centre.

Among the reported cases, 264 patients (hot: 144; cold: 123) presented within one day of referral, 190 (hot: 73; cold: 117) within one week, and 83 (hot: 53; cold: 30) after one week. Notably, hot cases were more likely to report earlier than cold cases (p = 0.001).

**Table 2** shows the results of the regression analysis, where a multivariable binary logistic regression model was used to identify the determinants of reporting rates. Variables related to the patient’s age and gender, the type of diagnosis, travel time from the VC to the hospital, and the number of years since the VC was established were included as covariates.

**Table 2:** Results of multivariable logistic regression.

Factor variable	Reference	Coefficient	Odds ratio (OR)	p-value
(Intercept)				
Gender of the patient				
Female	Male	-0.17	0.845	0.066
Age of the patient				
> 40 years	< 40 years	0.18	1.192	0.075
> 60 years	40–60 years	-0.07	0.937	0.599
Nature of diagnosis				
Hot	Cold	0.69	1.989	0.000*
Travel time				
Less than 30 mins	30–60 mins	0.89	2.426	0.000*
Greater than 60 mins	30–60 mins	0.51	1.667	0.000*
Years since establishment of the VC				
> 1 year	< 1 year	3.06	21.236	0.000*
> 3 years	1–3 years	0.58	1.785	0.000*
> 5 years	3–5 years	0.04	1.040	0.767
> 10 years	5–10 years	1.03	2.797	0.000*

Regression analysis showed a significantly higher likelihood of reporting among patients with potentially urgent or serious (hot) diagnoses compared to those with cold diagnoses (p < 0.001).

Travel time showed a non-linear relationship with reporting rates. Patients from VCs within 30 minutes of the hospital were more likely to report compared to those living 30–60 minutes away. Interestingly, reporting rates increased again among patients from VCs located more than an hour away. The higher likelihood of reporting in both the shortest and longest travel-time groups was statistically significant (p < 0.001 for both comparisons). Longer-established VCs were strongly linked to higher reporting. Compared to centres less than one year old, those older than one year had significantly higher odds of patient reporting (p < 0.001). VCs operating for more than 3 years had even higher odds (p < 0.001), and it increased significantly again in VCs operating for over 10 years (p < 0.001). The odds of reporting do not significantly change among VCs operating for 3–10 years (p = 0.767).

Female patients were slightly less likely to report than male patients, although this association was not statistically significant (p = 0.066). Patients over 40 years old showed a tendency to report more often than younger patients (p = 0.075), although this also did not reach the conventional 5% significance level.

## Discussion

Through this study, we aimed to evaluate the use of teleophthalmology to deliver specialist care for corneal diseases to VCs in remote areas of northern India, through a provider-to-provider, app-based consultation model. Patients were registered by trained VTs and remotely assessed by cornea specialists at a tertiary centre. Based on the consultation, patients were either managed locally or referred to higher levels of care. To our knowledge, this is the first study to report the management of corneal pathologies via teleconsultation by cornea specialists, thereby extending expert care to remote and resource-limited regions of the country.

Of the 62 teleophthalmology models identified during literature review, most studies utilised local care providers (e.g., nurses, optometrists, ophthalmic technicians, Indigenous health workers, general practitioners [GPs]) to consult an ophthalmologist at a distant location via teleophthalmology (12). In India, VTs posted at VCs form the backbone of the teleophthalmology infrastructure. They are the first point of contact for patients in rural areas (10). Teleophthalmology services in India largely rely on these VTs for providing appropriate primary care and referrals.

Managing patients at the VCs significantly reduces healthcare costs and the time burden for patients. Importantly, the ability to diagnose and treat corneal diseases at the primary care level represents a major advancement in delivering ophthalmic services. Without teleophthalmology, all such cases would have required referral to higher centres. By integrating telemedicine, 2, 022 patients—representing 42% of all those who received teleconsultations—were managed medically at the VC itself. Given a referral compliance rate of only 28%, it is estimated that, if these 4, 825 patients had been referred, only 1, 351 would have accessed care at a higher facility. This demonstrates the significant impact of teleophthalmology in improving access to timely, appropriate, and equitable eye care, while potentially preventing unnecessary referrals. This model not only ensured timely and accessible treatment but also helped optimise resource utilisation at referral centres by prioritising care for patients with more complex needs.

The most common acute corneal conditions we found included corneal ulcers, epithelial defects, corneal foreign bodies, viral keratitis, and corneal abrasions. In our study, 658 patients were diagnosed with corneal ulcers, of whom 103 patients with less than 1 mm size were successfully managed at the VC level. This demonstrates the feasibility of managing select cases of microbial keratitis at the primary care level with appropriate tele ophthalmic support. Evidence from previous studies has shown that simple, early interventions by allied eye health personnel can significantly reduce the incidence of microbial keratitis in rural settings. Research conducted in Burma, South India, Bhutan, and Nepal has demonstrated the effectiveness of topical antibiotics and antifungals in preventing bacterial and fungal keratitis following corneal trauma (13–16). Many of the acute cases were effectively treated at the primary care level, such as 77% of epithelial defects (n = 324), 81% of corneal abrasions (n = 57), and 85% of corneal foreign bodies (n = 380). Since these conditions make up the majority of urgent corneal issues, managing such a high volume at the VC level via teleophthalmology is a significant step forward in community-based eye care.

The application of telemedicine in the management of corneal pathologies facilitates timely diagnosis and intervention and significantly reduces the overall cost of care delivery. Rathi et al. assessed the economic implications of early treatment for corneal abrasions and ulcers across the LVPEI network. They demonstrated that prompt management at VCs and secondary centres (SCs) was markedly more cost-effective than delayed intervention at tertiary centres (TCs), with mean societal costs of INR 280, 11, 720, and 58, 850, respectively (17). Similarly, Shah et al. reported on the implementation of a VC-based teleophthalmology model for primary eye care delivery in South Gujarat, India (18). In their study, corneal diseases made up 5.2% of all cases—ranking after cataract and refractive errors—and the model proved highly cost-effective, saving an average of INR 621 per patient.

Supporting the scalability and impact of app-based teleophthalmology, Das et al. documented the largest and most widespread implementation through the eyeSmart EMR platform, deployed across 176 VCs in four South Indian states (19). Here, 63, 703 patients were evaluated, with 47.5% referred to higher centres and a reporting rate of only 19%. Corneal and anterior segment disorders were the most common diagnoses, accounting for 71.1% (n = 45, 261) of all cases—highlighting the critical role of teleophthalmology in the effective triage and management of anterior segment conditions at the primary care level.

Interestingly, in our study, the regression analysis showed a non-linear relationship between travel time and reporting rates. Patients living within 30 minutes of the referred hospital were more likely to report, probably due to easier access. Interestingly, reporting rates also rose among those living more than an hour away. This pattern might indicate limited access to local eye care services in more remote areas, which could increase referral compliance. These findings imply that both proximity and the availability of alternative healthcare options influence patient follow-through. This observation is similar to the results reported by Padhy et al., who studied barriers to referral uptake from primary to secondary centres and found that patients living farther from SC had higher reporting rates, possibly due to hospital-provided transportation (20).

Our analysis demonstrated a strong association between the duration of VC operation and patient reporting rates. Centres functioning for more than one year showed significantly higher odds of referral compliance compared to those established within the past year (p < 0.001), with the highest reporting rates observed at centres operating for over 10 years (p < 0.001). This pattern likely reflects the cumulative benefits of community trust, established patient–provider relationships, and more efficient referral pathways developed over time. Additionally, longer-standing centres may benefit from experienced staff, increased patient awareness, and a stable catchment population—factors that collectively enhance referral adherence.

The regression analysis also showed a slight trend toward lower referral compliance among female patients compared to males, although the association did not reach statistical significance (p = 0.066). This finding aligns with previous studies suggesting that sociocultural barriers, mobility constraints, and caregiving responsibilities may influence women’s healthcare-seeking behaviour, particularly in rural settings. Similarly, patients over the age of 40 were more likely to report than younger individuals (p = 0.075), possibly reflecting increased health awareness, perceived vulnerability, or prioritisation of vision in later life. While these associations were not statistically significant at the conventional 5% threshold, the observed patterns merit further investigation, especially in the context of targeted strategies to improve referral adherence across demographic groups.

This study has some limitations. Clinical photographs were taken with different smartphone cameras without standardization, which may have caused variability in image quality. Previous studies assessing anterior segment imaging across diverse devices have reported decreased diagnostic sensitivity, emphasizing this as a known challenge (21). Images were captured by multiple users (VTs), and standardized image acquisition protocols were not applied. However, since the main goal was rapid triage and early referral, strict image standardization was not a priority. The need for multicentre studies to evaluate image quality across different users and devices remains an important area for future research. Although protocols were established so that every case underwent teleconsultation, a few cases may have been missed and were not evaluated, as tracking these was beyond the scope of this study. Studying cost-effectiveness could have added financial perspective to the current study and could be a subject for future research in a similar setting.

## Conclusion

Teleconsultations with cornea specialists offer diagnostic accuracy and nuanced clinical judgement for managing both acute and non-acute corneal conditions at the primary level, specifically in rural and resource-limited areas. By combining image-based diagnosis with expert oversight, this approach improves the quality of care at the primary level while ensuring that patients requiring escalation are promptly and appropriately referred. In addition to ensuring quality of care, this approach can help create capacity at the hospital level by reducing unnecessary referrals.

## Data Availability

The raw data supporting the conclusions of this article will be made available by the authors, without undue reservation.
